# Be careful what you wish for: Individuals perceived to desire status are afforded less status

**DOI:** 10.1371/journal.pone.0304727

**Published:** 2024-06-25

**Authors:** Andrew L. Choi, Cameron Anderson

**Affiliations:** Haas School of Business, University of California, Berkeley, California, United States of America; University of Agder: Universitetet i Agder, NORWAY

## Abstract

In multiple studies, we found that people who are viewed as possessing a stronger desire for status are, ironically, afforded lower status by others. Coworkers who were viewed as having a higher (versus lower) desire for status (Study 1a and 1b), and individuals who were described as having a higher desire for status (versus a lower desire for status or no information), were afforded lower status (Studies 2, 3a, and 3b). Mediation analyses and an experimental manipulation of the mediator (Study 3a and 3b) suggested that the observed negative effect of desire for status on status was mediated primarily by perceptions of low prosociality. These findings have important implications for status organizing processes in groups.

## Introduction

Anthropologists have observed that in many traditional small-scale societies, such as the Navajo [1] and Zuni [2] peoples, individuals who are viewed as strongly desiring status are prohibited from occupying positions of authority because they are seen as less trustworthy. For example, groups will not appoint an individual to be leader if that person appears to desire the role too strongly. In the current research, we examined this idea empirically–specifically testing whether individuals who are viewed as desiring status are, ironically, afforded less status by others because they are viewed as being less prosocial and more selfish.

We tested these hypotheses in five studies that were all preregistered (total *N* = 1,811). These studies examined the effects of the perceived desire for status on status affordance in real-world groups (Studies 1a and 1b) and in online laboratory experiments that allowed for causal inference (Studies 2, 3a, and 3b). They also examined perceived prosociality as the primary mediating mechanism (Studies 1a, 1b, 2, 3a, and 3b).

### The desire for status

Status is defined as the respect, admiration, and voluntary deference individuals are afforded by others [3,4]. Status is akin to prestige in the dual pathways model (i.e., dominance versus prestige; [5,6]), and results in a plethora of benefits, such as greater influence and privileged access to resources. As such, the desire for high status is a fundamental human motivation [3,7,8], and yet, individuals differ in the strength of their status motive. Some individuals desire status more strongly than others and work harder to attain it [9–11].

How do individuals attain higher status? An abundance of evidence has shown that groups afford high status to individuals who are perceived to provide instrumental social value [12], that is, individuals who appear to possess personal characteristics that will facilitate the group’s success [13–17]. While the personal characteristics seen as providing value to a group can vary across groups and depend on their specific goals, in general groups afford higher status to individuals when they appear to possess two kinds of personal characteristics.

First, individuals must seem to possess competencies that are central to the group’s primary tasks and challenges [18,19]. For instance, in a task-oriented group (e.g., workgroup), valued competences may include the technical skills necessary to complete difficult problems. Second, individuals must appear prosocial, in that they seem willing to use their competence to help the group succeed [20–22]. Altruistic and generous behavior, especially when viewed as authentic, drives status affordance [23]. As Blau [14] explained, “To earn the deference as well as the respect of others, it is not enough for an individual to impress them with his outstanding qualities; he must use these abilities for their benefit” (p. 162). Therefore, highly competent individuals who are capable of facilitating the group’s goals will only attain high status if they are also viewed as prosocial, or willing to facilitate the group’s goals [24,25].

**Fig 1:**
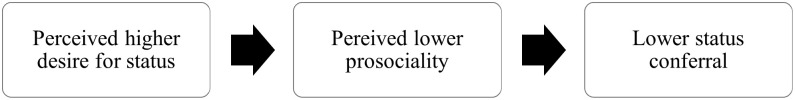
Our proposed model.

Based on the above model, we hypothesized individuals perceived as possessing a stronger desire for status will be afforded lower status, because they will be seen by others as less prosocial and more selfish (see Fig 1). Prima facie, the desire for status involves seeking benefits for oneself, such as social attention, admiration, and influence [26]. In this way, the desire for status is characterized as a self-enhancement concern as opposed to a more self-transcendent value such as benevolence [27]. Further, prior research suggests extrinsic (vs. intrinsic) motives tend to be viewed as more selfish [28]. Indeed, Kim and Pettit [29] found that people viewed the desire for status negatively and attempt to hide their own desire for status from others. Their findings are consistent with our contention that individuals seen as desiring status more strongly will be afforded lower status. Accordingly, we propose the following hypotheses:

***H1***: *Individuals perceived to have a higher desire for status will be conferred lower status than individuals perceived to have a lower desire for status*.
***H2***: *The negative effect of perceived desire for status on status affordance will be mediated by perceptions of prosociality*.

We do not have any specific hypotheses regarding perceptions of competence, the other precursor to status mentioned above. While perceived competence is important to status organizing processes in groups, we know of no prior theory or research that suggests individuals who desire status more strongly will be viewed as less competent. Therefore, we examined perceived competence along exploratory lines.

### Prior research

To our knowledge, only one prior investigation [23] has examined the effect of the perceived desire for status on status affordances, and it found a positive rather than negative effect. However, its measure of the desire for status included whether the person sought social approval in addition to whether they desired others’ respect. Therefore, it is possible the positive effect they observed emerged because they combined the effects of the perceived desire for acceptance with the perceived desire for status. In our studies, we manipulate or measure the desire for status per se, without including other social outcome variables.

## Study 1a

Study 1a focused on people’s perceptions of their actual coworkers. We hypothesized that participants would accord lower status to coworkers they viewed as possessing a higher desire for status. We also tested whether coworkers perceived as higher in the desire for status would be afforded lower status because they lacked prosociality. Along exploratory lines, we measured perceived competence, as well as the length and type of relationship (i.e., boss, peer, or subordinate) between the participant and the coworkers being rated; including the latter measures allowed us to rule them out as possible third variables.

### Methods

We preregistered our study on AsPredicted (http://aspredicted.org/blind.php?x=v2zw3q). This research has been approved by the Institutional Review Board at the University of California, Berkeley. Specifically, the protocol has been approved by the Committee for Protection of Human Subjects at UC Berkeley (Protocol Number: 2019-03-11890).

#### Participants

A total of 169 participants (58.5% male, 41.5% female) with an average age of 38.29 (*SD* = 9.19) were recruited from Amazon Mechanical Turk. Using the simr package in R [30], to detect a negative correlation of -.20, it was estimated that we would have 91.90% power with 169 participants. Given that the study design involved questions about coworkers, participants were required to be employed full-time or part-time to participate. All participants completed identical surveys in which they rated their coworkers and were paid $1.40 for an approximately 8-minute survey. Five participants failed at least one of two pre-registered attention checks, resulting in 164 total participants. All participants provided written informed consent. The recruitment period for this study began and ended on August 26^th^, 2019.

#### Procedure

Participants first nominated seven coworkers they would rate in this study. In particular, we told participants the following information:

“Please think of 7 coworkers from your workplace. These should be people who you work with at least somewhat closely (as opposed to someone you rarely see or work with). Below, please list the initials of these 7 coworkers, making sure that each coworker has a unique set of initials, different from everyone else’s.”

To help ensure that the coworkers they rated were randomly selected, rather than only their closest coworkers, we randomly selected three of the seven coworkers participants nominated. Participants then rated those three coworkers, in random order, on the measures below.

### Materials (survey)

Unless otherwise noted, all scales were rated from 1 (“Strongly disagree”) to 7 (“Strongly agree”).

#### Independent variable: Coworker’s desire for status

Participants rated their coworker’s desire for status with three items adapted from a widely used scale (Flynn et al., 2006): “(*Initials*) is highly concerned with having high social status”; “It would please (*Initials*) to have a position of prestige and social standing”; and “(*Initials*) does not care about his status among peers” (reverse-scored). The items showed internal consistency (*α* = .74) and were combined (*M* = 4.24, 95% CI = [4.09, 4.38]).

#### Dependent variable: Affordance of status

Status conferral was measured with three items that reflect the definition of status: “I respect (*Initials*)”; “I admire (*Initials*)”; and “I grant status to (*Initials*).” The items showed internal consistency (*α* = .77) and were combined (*M* = 4.79, 95% CI [4.65, 4.92]). We used peer-ratings of status as the primary measure of status affordance because status “lives” in the minds of others and is grounded in their respect and admiration of a person; furthermore, peer-ratings of status correspond with other status proxies such as influence [31] and the attainment of leadership positions [32].

#### Mediator: Prosociality

Participants rated their coworkers’ prosociality with six items, consisting of three items from prior scales [33] and three additional items: self-interested (reverse coded), altruistic, moral, principled, warm-hearted, and honest. The items showed internal consistency (*α* = .81) and were combined (*M* = 4.98, 95% CI [4.86, 5.10]).

#### Additional measures

Participants also rated their coworkers’ competence with three items from a widely used scale [31]: capable, competent, and skillful. The items showed internal consistency (*α* = .92) and were combined (*M* = 5.75, 95% CI [5.63, 5.86]). Moreover, to account for potential confounds, participants reported how long they had worked with each of their coworkers (*M* = 4.92 years, *SD* = 4.71). Participants also reported the type of relationship they had with their coworkers, indicating whether they reported to their coworker (25.6%), or whether their coworker reported to them (17.3%), or neither (57.1%).

### Results and discussion

Ratings of coworkers were nested within participants, so we analyzed our data using mixed-effects modeling that included random intercepts and slopes for participant. As predicted, there was a negative relationship between perceptions of desire for status and status affordance (*β* = -0.12, 95% CI = [-.21, -.03], *t*(131.49) = -2.602, *p* = .010), and between desire for status and perceived prosociality (*β* = -0.29, 95% CI = [-0.36, -0.21], *t*(121.89) = -7.629, *p* < .001), indicating participants afforded lower status and viewed as less prosocial those coworkers they saw as possessing a stronger desire for status. There was no relationship between the perceived desire for status and perceived competence (*β* = -0.07, 95% CI = [-.15, .01], *t*(122.30) = -1.772, *p* = .079).

**Fig 2:**
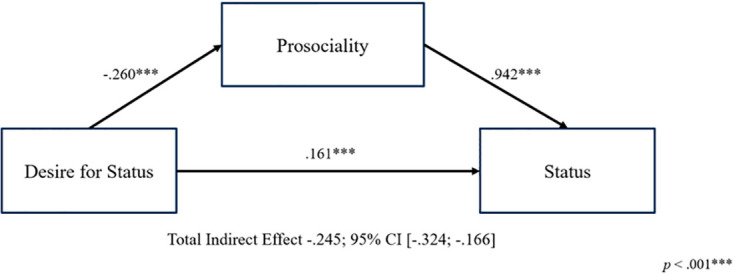
Prosociality mediation model for Study 1a.

We next conducted mediation analyses through the Lavaan package in R [34], which allows for the mediation to account for the nested nature of the data. As predicted, perceived prosociality mediated the negative relationship between the perceived desire for status and the affordance of status -.245, (95% CI = [-.324, -.166]; Fig 2). A simultaneous mediation including both prosociality and competence again revealed a significant indirect effect of prosociality, -.166 (95% CI:—.225, -.107). In contrast, perceived competence had a non-significant indirect effect of -0.028 (95% CI:—.065, .010). These findings suggest that participants afforded lower status to coworkers they viewed as desiring status because they viewed those coworkers as less prosocial, but not because they viewed those coworkers as less competent.

In addition, we found that the negative relationship between perceived desire for status and status held up even when controlling for relationship length and the type of relationship participants had with their coworker (*β* = -0.14, 95% CI = [-.23, -.05], *t*(133.04) = -3.021, *p* = .003). Therefore, the negative relationship between perceived desire for status and status was not due to potential confounding variables such as relationship length and the target’s hierarchical level vis-à-vis the participant.

Along exploratory lines, we also tested for a curvilinear effect to see whether a low desire for status enhanced status, a high desire for status dampened status, or both. In regression analyses with both linear and squared terms for perceived desire for status, we found that the squared term had a significant and negative effect (*β* = -0.12, 95% CI = [-.18, -.06], *t*(419.65) = -3.918, *p* < .001). The linear term was also significant and negative (*β* = -0.16, 95% CI = [-.26, -.06], *t*(99.77) = -3.235, *p* = .002). The significant squared term suggests that the effect of perceived desire for status was curvilinear. We next conducted a tertiary split of the perceived desire for status. In one-way ANOVAs, we found coworkers with the highest perceived desire for status were afforded significantly less status (*M* = 4.46, 95% CI = [4.20, 4.72]) than coworkers with a moderate desire for status (*M* = 4.92, 95% CI = [4.67, 5.17], *p* = .016), and coworkers with the lowest desire for status (*M* = 4.93, 95% CI = [4.70, 5.16], *p* = .007). There were no significant differences between coworkers in the lowest third and the coworkers in the middle third, *p* = .999. This pattern of results suggests that the negative effect of perceived desire for status was strongest at the highest level of the perceived desire for status.

## Study 1b

The goals of Study 1b were to directly replicate Study 1a with twice the number of participants and a different participant pool, and to conduct a well-powered test of the curvilinear effect found in Study 1a, given the finding was unexpected. With the larger sample size, it was estimated that Study 1b would be powered at 99.90% (95% CI [99.44, 100.00]) to test the curvilinear effect found in Study 1a (analysis conducted via the simr package in R).

### Methods

We pre-registered the study on AsPredicted (https://aspredicted.org/blind.php?x=7jw422). This research has been approved by the Institutional Review Board at the University of California, Berkeley. Specifically, the protocol has been approved by the Committee for Protection of Human Subjects at UC Berkeley (Protocol Number: 2019-03-11890).

#### Participants

A total of 352 participants (50.9% male, 48.0% female, 1.1% other) with an average age of 33.41 (*SD* = 10.07) were recruited from Prolific Academic. Given that the study design involved questions about coworkers, participants were required to be employed full-time or part-time, and working more than 10 hours each week to participate. All participants completed identical surveys in which they rated their coworkers and were paid $1.40 for an approximately 8-minute survey. All participants provided written informed consent. The recruitment period for this study began and ended on January 25^th^, 2021.

#### Procedure

The procedure was identical to the procedure from Study 1a with two exceptions. First, participants reported the gender (49.6% male, 50.3% female, .01% other) and age of the coworker they were rating (*M* = 37.97, *SD* = 12.58). We used the same scales for perceived desire for status (*M* = 4.29, 95% CI = [4.20, 4.37]), affordance of status (*M* = 4.77, 95% CI [4.69, 4.85]), perceived prosociality (*M* = 4.97, 95% CI [4.90, 5.04]), and perceived competence (*M* = 5.62, 95% CI [5.54, 5.69]), all of which again demonstrated acceptable reliability (*αs* = .75, .75, .87, and .93, respectively).

### Results and discussion

Replicating our findings from Study 1a, there were negative relationships between the perceived desire for status and status affordance (*β* = -0.14, 95% CI = [-.21, -.08], *t*(192.24) = -4.194, *p* < .001), and between the perceived desire for status and perceived prosociality (*β* = -0.31, 95% CI = [-0.36, -0.25], *t*(207.29) = -10.92, *p* < .001). In contrast to Study 1a, participants also viewed coworkers higher in the desire for status as less competent (*β* = -0.12, % CI = [-0.18, -0.05], *t*(240.43) = -3.497, *p* < .001).

We next conducted mediation analyses through the Lavaan package in R [34]. As predicted, perceived prosociality mediated the negative relationship between the desire for status and affordance of status. The analysis revealed an indirect effect of -.182 (95% CI:—.223, -.141), indicating a significant indirect effect. Unexpectedly, competence also had a significant indirect effect of -.044 (95% CI:—.073, -.016). However, a pairwise contrast test of indirect effects indicated that the indirect effect of prosociality was significantly stronger, -.138 (95% CI: -.176, -.101) than the indirect effect of competence. These findings suggest that participants afforded lower status to coworkers they viewed as strongly desiring status primarily because they viewed those coworkers as less prosocial.

Along exploratory lines, we tested whether the gender of the coworker moderated the relationship between perceived desire for status and status conferral. Based on prior work suggesting that ambition in women (versus men) incurs more backlash, we explored whether perceptions of the desire for status would result in lower status conferrals for women than for men [35,36]. We found a significant interaction between perceived desire for status and the gender of the coworker on status conferral (*β* = .17, 95% CI = [.06, .27], *t*(945.66) = 3.074, *p* = .002). In line with the prior work on backlash, the negative relationship between perceived desire for status and status conferral was greater for female coworkers, *β* = -.25, *t*(966) = -6.327, *p* < .001, than male coworkers, *β* = -.09, *t*(958) = -2.260, *p* = .024. Therefore, the negative relationship between desire for status and status affordance was observed for both women and men, but the effect was stronger for women (versus men). We also tested for an interaction effect between age and perceived desire for status on status conferral. No interaction effect between perceived desire for status and age was found. Finally, we found that the main effects of perceived desire for status remained robust even after controlling for the age and gender (*β* = -0.14, 95% CI = [.-.21, -.08], *t*(196.26) = -4.316, *p* < .001) of the coworkers being rated.

The inverted-U curvilinear effect found in Study 1a was not replicated with the larger sample size. In regression analyses with both linear and squared terms for perceived desire for status, we found that the squared term had a null effect (*β* = -0.04, 95% CI = [-.09, .00], *t*(530.60) = -1.78, *p* = .076) on status conferral. The linear term remained significant and negative (*β* = -0.21, 95% CI = [-.28, -.14], *t*(136.06) = -5.99, *p* < .001).

## Study 2

Study 2 had several aims. First, we sought to establish the causal effect of perceived desire for status by manipulating whether or not a target possessed a strong desire for status. Second, we tested whether targets with a strong a desire for status would be afforded lower status than others even when they engage in similar behavior as those others. If so, this would suggest that even when individuals engage in identical actions as other people do, they will be afforded lower status if their behavior is attributed to the desire for status. Third, given the gender moderation effect we found in Study 1b, along exploratory lines, we wanted to test for such moderation using an experimental design.

Participants first read a standardized introduction about a target’s characteristics and behavioral patterns (e.g., a strong work ethic). However, participants then learned that the target’s behavior was driven by either the desire for status or the desire for achievement. The desire for achievement was chosen as the relevant comparison because both motives can spur similar behaviors (e.g., working hard) and the desire for achievement is also considered a fundamental motivation [37,38]. A control condition was also included, in which an individual was described without any explicit mention of underlying desires. We predicted that the target described as possessing the desire for status would be afforded less status than targets possessing the desire for achievement or with no desire information.

### Methods

The study was preregistered on OSF (https://osf.io/9t83s/?view_only=370d0c8242ad4e). This research has been approved by the Institutional Review Board at the University of California, Berkeley. Specifically, the protocol has been approved by the Committee for Protection of Human Subjects at UC Berkeley (Protocol Number: 2019-03-11890).

#### Participants and design

A total of 606 adult participants (53.5% male, 46.5% female) with an average age of 36.38 (*SD* = 9.73) were recruited from Amazon Mechanical Turk. We predetermined the target sample size to be 600 to allow for 100 participants per condition, with consideration of the minimum sample size per condition recommended by previous research (50 per condition; [39]), but also recognizing that the study was conducted online. Participants were paid $.50 for an approximately 3-minute survey. The study had a 3 (desire: desire for status, desire for achievement, control) x 2 (target gender: male, female) between-subjects design. All participants provided written informed consent. The recruitment period for this study began and ended on April 17, 2019.

#### Procedure

Participants read about a person named Kevin or Katherine and were asked to imagine they had been working with that person. All participants first learned about the target’s characteristics, which included a strong work ethic, a pleasant demeanor, a willingness to make sacrifices for the organization, and emotional stability (see S1 Appendix for complete materials). However, the motivations underlying these characteristics were then experimentally manipulated. These manipulations were built from prior research [40,41]. In the high desire for status condition, participants read:

“You have also learned over time how much Kevin’s behavior is driven by his desire for status–that is, he is deeply concerned about being highly respected and admired by those around him, and being influential. He works hard in part because he cares so much about winning others’ esteem. While he is helpful with coworkers, he is particularly willing to help if others will find out about his sacrifices. For example, he is much more likely to work longer hours to help his team if he believes it will make others admire him more, and if he believes that his efforts are being seen. Even his happiness and self-esteem seem to depend on whether he feels respected and admired by his coworkers.”

In contrast, in the high desire for achievement condition, his/her behavior was driven by a desire for achievement (e.g., “He works hard in part because he cares so much about achieving”):

“You have also learned over time how much Kevin’s behavior is driven by his desire to achieve–that is, he is deeply concerned about accomplishing great things and performing as well as he can in all facets of life, including work. He works hard in part because he cares so much about achieving. While he is helpful with coworkers, he is particularly willing to help if it means he will feel a sense of accomplishment. For example, he is much more likely to work longer hours to help his team if he believes it will lead to higher performance and important accomplishments. His happiness and self-esteem seem to depend on whether he feels as though he is performing well at work.”

In the control condition, participants read a neutral description that omitted information about the target’s underlying desires or motivations. After participants finished reading about the target, they rated the target on dimensions described below.

### Materials (survey)

#### Status conferred to target

With the same measures as in Studies 1a and 1b, participants rated the extent to which they would afford the target status (e.g. “I would grant status to Kevin,” “I respect Kevin,” “I admire Kevin”), on a scale from 1 (“Strongly disagree”) to 7 (“Strongly agree”). The items showed internal consistency (*α* = .88) and were combined (*M* = 5.53, 95% CI [5.44, 5.62]).

#### Prosociality and competence

Participants rated their target’s prosociality with the same six items used in Studies 1a and 1b, in addition to two items (“Kevin is trustworthy,” “Kevin is genuine”), rated from 1 (“Strongly disagree”) to 7 (“Strongly agree”). The items showed internal consistency (*α* = .92) and were combined (*M* = 5.06, 95% CI [4.97, 5.14]). Competence was measured with the same three items used in Studies 1a and 1b, in addition to two items (“Kevin is efficient,” “Kevin is intelligent”). The five items showed internal consistency (*α* = .89) and were combined (*M* = 6.11, 95% CI [6.05, 6.18]).

### Results

**Fig 3:**
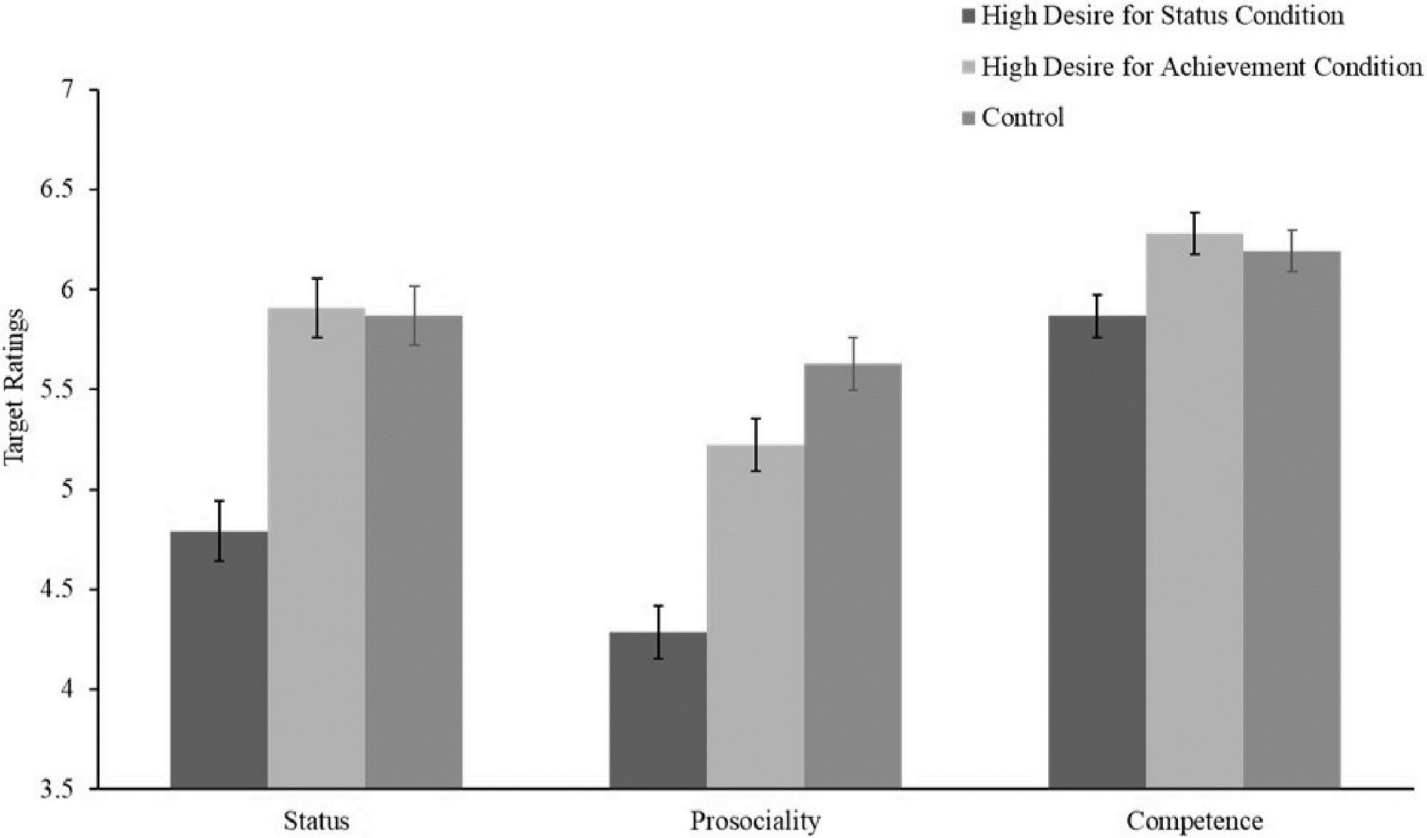
Study 2. Ratings by condition. 95% CIs shown.

As hypothesized, we found a significant main effect of the desire for status on status affordance, *F*(2, 600) = 71.6, *p* < .001. As shown in Fig 3, targets described as high in the desire for status (*M* = 4.79, 95% CI [4.60, 4.98]) were afforded significantly less status than targets described as high in the desire for achievement (*M* = 5.91, 95% CI [5.78, 6.03], *t*(341.723) = 9.527, *p* < .001, d = .957), and control targets (*M* = 5.87, 95% CI [5.76, 5.99], *t*(319.664) = 9.538, p < .001, *d* = .958). Two-way ANOVAs revealed no significant interactions between the desire for status and gender in affecting status or any other variable, indicating that the effect of desire for status on status affordance, prosociality, and competence was not moderated by the target’s gender (status: *F*(2, 600) = .475, *p* = .622, prosociality: *F*(2, 600) = 2.095, *p* = .124, competence: *F*(2, 600) = 2.270, *p* = .104).

A significant main effect of condition on prosociality perceptions was also found, *F*(2, 600) = 106.868, *p* < .001. Participants rated targets described as high in the desire for status (*M* = 4.29, 95% CI [4.15, 4.42]) as less prosocial than targets described as high in the desire for achievement (*M* = 5.22, 95% CI [5.09, 5.35], *p* < .001), and control targets (*M* = 5.63, 95% CI [5.50, 5.76], *p* < .001). These results replicate the prosociality findings in Studies 1a and 1b. A similar main effect was found for competence perceptions, *F*(2, 600) = 16.993, *p* < .001. Participants rated targets described as high in the desire for status (*M* = 5.87, 95% CI [5.81, 5.92]) as less competent than targets described as high in the desire for achievement (*M* = 6.28, 95% CI [6.23, 6.33], *p* < .001), and control targets (*M* = 6.19, 95% CI [6.14, 6.24], *p* < .001).

To examine the role of prosociality and competence perceptions in the affordance of status, we used Preacher and Hayes’s bootstrapping procedure [42], 5000 resamples with replacement to derive a 95% bias-corrected confidence interval for the indirect effects of the desire for status on status. Focusing on the comparison between the control condition and the high desire for status condition, a simultaneous mediation revealed an indirect effect for prosociality of -.7940 (95% CI:—.9411, -.6490), indicating a significant indirect effect. Competence also had a significant indirect effect of -.1044 (95% CI:—.1718, -.0496). However, a pairwise contrast test of indirect effects indicated that similar to Study 1b, the indirect effect of prosociality was significantly stronger, -.6896 (95% CI: -.8556, -.5160), than the indirect effect of competence. These findings suggest that participants afforded lower status to targets described as having a high desire for status primarily because they viewed those targets as less prosocial.

## Study 3a

Study 3a had two primary aims. First, it aimed to rule out an alternative explanation for the findings in Study 2: Namely, participants might have inferred that the target described as having a higher desire for status already had lower status in his/her workplace and afforded low status to him/her accordingly. Perhaps participants held an implicit belief that having low status boosts the desire for status, and thus inferred that the individual who desired status strongly must have had low status already. We aimed to eliminate this alternative explanation by describing the targets as being new employees in a company and thus as not having any preexisting status in the company, holding constant the individual’s hierarchical standing and characteristics besides the status motive.

Second, Study 3a aimed to provide further evidence that perceptions of low prosociality underlie the effects of the desire for status on the affordance status, by experimentally manipulating the prosociality of the target [43]. Specifically, we manipulated whether the target’s desire for status was driven by prosocial or selfish concerns; it seems reasonable that at least for some individuals, they desire status so they can use their lofty position for the good of others. Accordingly, we hypothesized that the negative effect of the perceived desire on status affordance would be reduced in the prosocial (as compared to selfish) condition, because the target with a prosocial intent—though high in the desire for status—would desire the status not for personal gain, but for the benefit of others.

### Methods

We preregistered this study on AsPredicted (https://aspredicted.org/blind.php?x=ee6xg2). This research has been approved by the Institutional Review Board at the University of California, Berkeley. Specifically, the protocol has been approved by the Committee for Protection of Human Subjects at UC Berkeley (Protocol Number: 2019-03-11890).

#### Participants and design

A total of 300 participants (58.9% male, 41.1% female) with an average age of 38.31 (*SD* = 11.67) were recruited from Amazon Mechanical Turk. The target sample size of 300 was decided to allow for 100 participants per condition, with consideration of the minimum sample size per condition recommended by previous research (50 per condition, [39]), but also recognizing that the study was conducted online. Participants were paid $.50 for an approximately 3-minute survey. The study had a 3-cell between-subjects design: 1) high desire for status with a low prosociality orientation, 2) high desire for status with a high prosociality orientation, and 3) control (no desire for status or prosociality information presented). Three participants failed a preregistered attention check (asking participants to select “Slightly agree” on the attention check question), resulting in 297 total participants. All participants provided written informed consent. The recruitment period for this study began and ended on June 10^th^, 2020.

#### Procedure

Participants were asked to imagine they were a new hire attending a new employee orientation and learning about another new employee (Kevin). The descriptions of the target were similar to those used in Study 2, and in the two desire for status conditions, participants read that much of the new employee’s behaviors are driven by their desire for status (e.g., “He works hard in part because he cares so much about winning others’ esteem”). However, in those two conditions the desire was described as either selfish or prosocial. In the selfish desire for status condition participants read:

“You also learn in a conversation that Kevin’s strong desire for status roots from his belief that if he attains high status, he will be better equipped to help himself reach his own personal career goals. In other words, Kevin desires high status because he believes having high status will help him succeed as an individual and will bring him the trappings of having high status.”

In the prosocial desire for status condition participants read:

“You also learn in a conversation that Kevin’s strong desire for status roots from his belief that if he attains high status, he will be better equipped to help his team and the company overall. In other words, Kevin desires high status because he believes having high status will help him be a high-contributing member of the team, allowing the team and the company to succeed.”

The control condition was identical to Study 2, except that the description of the target omitted any discussion of underlying desires or motivations. Participants then rated the target on the dimensions below.

### Materials (survey)

#### Status conferred to target

Status was measured by an identical three-item measure used in Study 2 (1 = *strongly disagree*, 7 = *strongly agree*; *α* = .89; *M* = 4.69, 95% CI [4.61, 4.77]).

#### Prosociality and competence

Prosociality (*M* = 4.37, 95% CI [4.31, 4.44]) and competence (*M* = 5.62, 95% CI [5.56, 5.68]) were measured using the same scales as in Study 2 (1 = *strongly disagree*, 7 = *strongly agree; α*s = .92 and .92).

### Results and discussion

**Fig 4:**
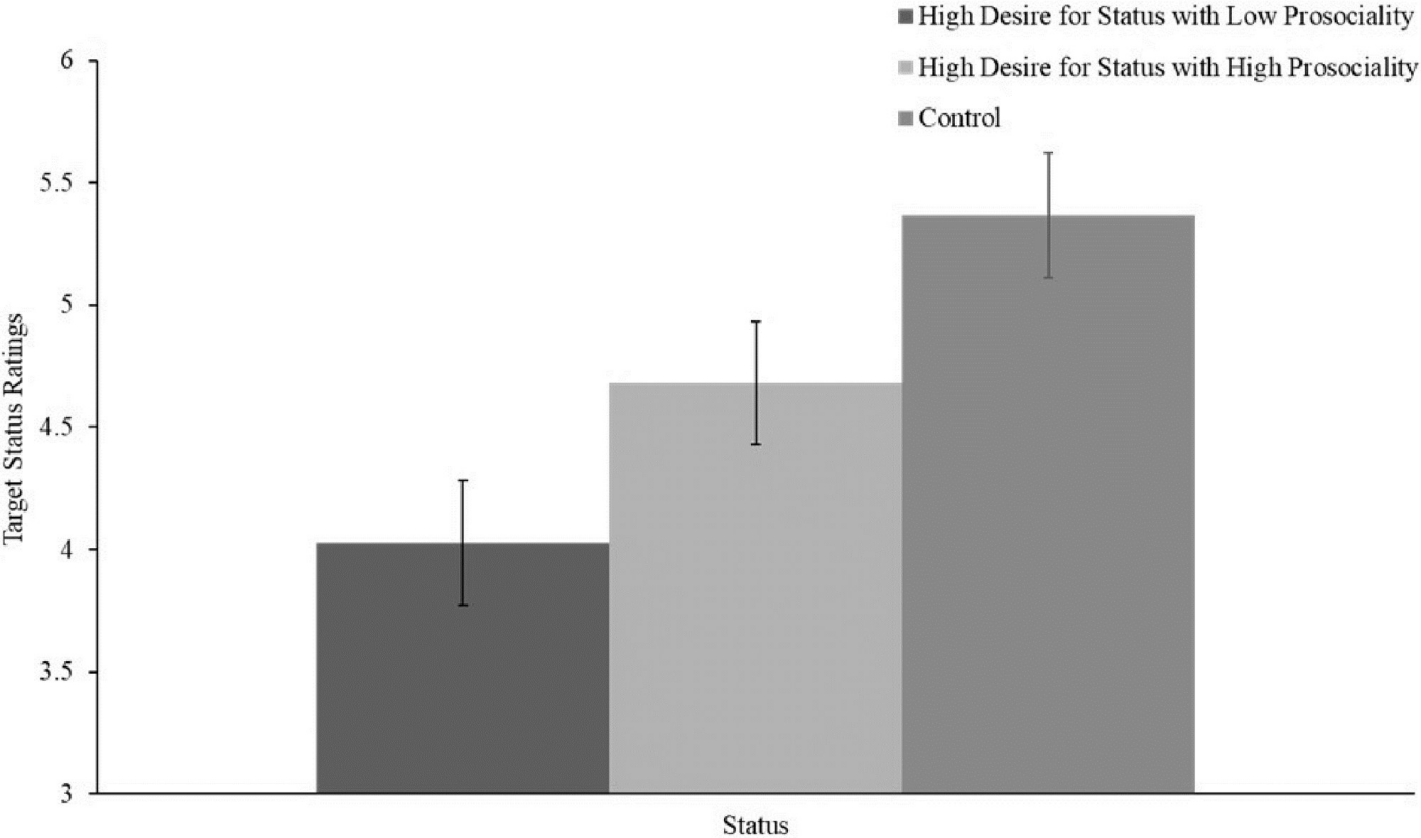
Status ratings by condition. 95% CI shown.

A one-way between subjects ANOVA revealed a significant effect of experimental condition on status conferral, *F*(2, 294) = 26.745, *p* < .001. Targets described as desiring status for selfish (i.e., non-prosocial) reasons (*M* = 4.03, 95% CI [3.90, 4.16]) were afforded significantly less status than targets described as desiring status for prosocial reasons (*M* = 4.68, 95% CI [4.55, 4.81], *p* < .001, *d* = .450), or targets in the control condition (*M* = 5.37, 95% CI [5.24, 5.50], *p* < .001, *d* = 1.162; see Fig 4). In addition, targets described as desiring status for prosocial reasons were also afforded significantly less status than targets in the control condition (*p <* .001, *d* = .562).

A significant main effect of experimental condition on prosociality perceptions was also found, *F*(2, 294) = 57.463, *p* < .001. Targets described as desiring status for selfish reasons (*M* = 3.61, 95% CI [3.41, 3.80]) were seen as being significantly less prosocial than targets described as desiring status for prosocial reasons (*M* = 4.38, 95% CI [4.19, 4.58], *p* < .001), or the control targets (*M* = 5.13, 95% CI [4.94, 5.33], *p* < .001). The difference between the targets desiring status for prosocial reasons and the control targets was also significant, *p <* .001. Further, a significant main effect of experimental condition on competence perceptions was found, *F*(2, 294) = 6.583, *p* = .002. Targets described as desiring status for selfish reasons (*M* = 5.36, 95% CI [5.16, 5.56]) were seen as being significantly less competent when compared to targets in the control condition (*M* = 5.89, 95% CI [5.69, 6.10], *p* < .001). However, targets described as desiring status for selfish reasons and targets described as desiring status for prosocial reasons (*M* = 5.62, 95% CI [5.42, 5.82]) were not significantly different in their competence ratings, *p* = .074. The competence ratings for the targets desiring status for prosocial reasons and the control condition did not significantly differ either, *p* = .063.

To further understand the role of prosociality and competence in the affordance of status, we again used Preacher and Hayes’s bootstrapping procedure [42], 5000 resamples with replacement to derive a 95% bias-corrected confidence interval for the indirect effects of desire for status on status, focusing specifically on the low prosociality desire for status condition and the control conditions. This analysis revealed an indirect effect of -.3862 (95% CI:—.4915, -.2970) for prosociality, indicating a significant indirect effect. Competence also had a significant indirect effect of -.0803 (95% CI:—.1314, -.0345). However, a pairwise contrast test of indirect effects indicated that the indirect effect of prosociality was significantly stronger, -.3059 (95% CI: -.5622, -.3752), than the indirect effect of competence. While the significant competence mediation results are not directly in line with the mediation results from Study 1a, the contrast test of the indirect effects suggests that the desire for status has a greater indirect effect on status through perceived prosociality than through perceived competence.

In addition, simultaneous mediation analyses focusing on the two desire for status conditions (i.e., high prosociality desire for status condition versus low prosociality desire for status condition) similarly revealed an indirect effect of -.6323 (95% CI:—.9070, -.3756) for prosociality, indicating a significant indirect effect. Competence did not have a significant indirect effect -.0955 (95% CI:—.2289, .0210). A pairwise contrast test also confirmed that the indirect effect of prosociality was significantly stronger, -.5368 (95% CI: -.7922, -.3194), than the indirect effect of competence.

These findings replicated the negative effect of perceived desire for status on status affordance, but the different setting described, in which targets are attending an employee orientation with no previous status hierarchy, helps refute an alternative explanation for the findings in Study 2: namely, that participants inferred targets high in the desire for status must have already had low status in the workplace. Furthermore, though the the prosociality manipulation did not completely eliminate the negative interpersonal effect of desiring status, the manipulation did mitigate it, consistent with our predictions. This provides further support that perceptions of prosociality underlie the negative relationship between perceived desire for status and status affordance.

## Study 3b

**Fig 5:**
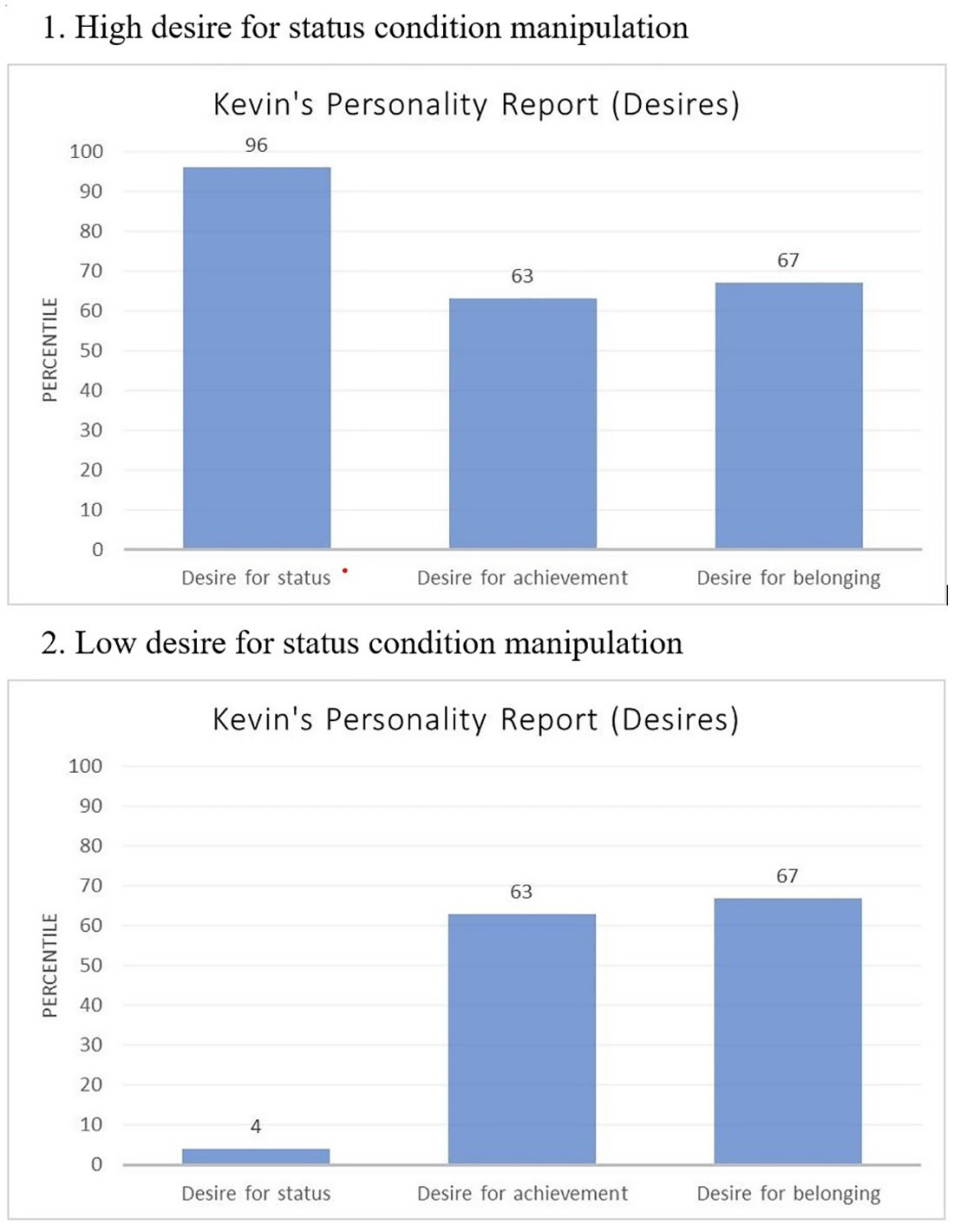
Visual plots of personality.

Study 3b aimed to rule out two potential alternative explanations for the findings in Study 3a. First, to help rule out the possibility that something in the verbal descriptions in Study 3a, other than the desire for status per se, drove our hypothesized effects, we provided participants with only a visual plot of the targets’ characteristics rather than a verbal description (see Fig 5). This would ensure that the manipulation focused solely on the targets’ desire for status and prevent the participants from assuming other negative traits of a target based on wordings such as “deeply concerned” (which may bring about assumptions of neuroticism; [44]) used in previous studies. Second, to help rule out the possibility that the high-desire conditions in Study 3a simply made the desire for status more salient than in the control condition–and this differential salience led to our effects, Study 3b compares high desire for status to those with a low desire for status, rather than to a control condition. Therefore, the desire for status would be salient in all conditions.

Furthermore, for the manipulation of prosociality, rather than describe an individual with low prosociality (as in Study 3a), Study 3b included a condition where no information about the individual’s prosociality was provided. In this way, we could assess the degree to which learning about individuals’ prosociality mitigates the backlash against them when they have a high desire for status (the high desire for status, prosociality condition) compared to simply inferring their level of prosociality from their desire for status (the high desire for status, no information about prosociality condition). We predicted that targets described as possessing a high (vs. low) desire for status would be afforded lower status; consistent with our mediation predictions, we further expected the negative effect of high (vs. low) desire for status to be mitigated when the target is described as high in prosociality.

### Methods

The study was preregistered on AsPredicted (https://aspredicted.org/blind.php?x=uw4w4x). This research has been approved by the Institutional Review Board at the University of California, Berkeley. Specifically, the protocol has been approved by the Committee for Protection of Human Subjects at UC Berkeley (Protocol Number: 2019-03-11890).

#### Participants and design

A total of 400 participants were recruited from Amazon Mechanical Turk, which would allow for 100 participants per cell, and be over the sample size per condition recommended by previous research (50 per condition, [39]), but also recognizing that the study was conducted online. The study had a 2 (high or low desire for status) x 2 (high prosociality or control) between-subjects design. The experimental conditions corresponded to 1) high desire for status target with high prosociality, 2) low desire for status target with a high prosociality, 3) high desire for status target with no information about their prosociality, and 4) low desire for status target with no information their prosociality. Thirteen participants failed at least one of two preregistered attention checks (asking participants to select “Slightly agree” on the attention check question), resulting in 387 total participants. All participants provided written informed consent. The recruitment period for this study began and ended on November 5^th^, 2020.

#### Procedure

As in Study 3a, participants were asked to imagine being a new hire attending a new employee orientation. At the orientation, another new employee (Kevin) shares his personality report that was part of the orientation. In the high desire for status conditions, the report stated that Kevin “placed at the 96th percentile on the desire for status (i.e., meaning he had a stronger desire for status than 96% of the population), suggesting Kevin has a very strong desire for status.” In the low desire for status conditions, the report stated that Kevin “placed at the 4th percentile on the desire for status (i.e., meaning he had a stronger desire for status than 4% of the population), suggesting Kevin has a very weak desire for status.” In the high prosociality conditions, participants also viewed a portion of the report stating that Kevin “placed at the 90^th^ percentile on his team-orientation, meaning that he is very team-oriented.” In the conditions with no information about prosociality, participants did not see any materials related to prosociality. After reviewing the relevant materials, participants rated the target on the dimensions below.

### Materials (survey)

#### Status conferred to target

Status was measured by an identical three-item measure used in Studies 2 and 3a (1 = *strongly disagree*, 7 = *strongly agree*; *α* = .82; *M* = 4.91, 95% CI [4.79, 5.04]).

#### Prosociality and competence

Prosociality (*M* = 4.76, 95% CI [4.65, 4.88]) and competence (*M* = 5.65, 95% CI [5.56, 5.74]) were measured using the same scales as in Study 3a (1 = *strongly disagree*, 7 = *strongly agree; α*s = .88 and .87).

### Results and discussion

Again replicating the negative effect of desire for status on status conferral, targets described as having a high desire for status (*M* = 4.53, 95% CI [4.35, 4.72]) were afforded significantly less status than targets described as having a low desire for status (*M* = 5.31, 95% CI [5.16, 5.45], *t*(367.565) = 6.514, *p* < .001, *d* = .659). Moreover, as expected from previous research and theory, targets described as being highly prosocial (*M* = 5.21, 95% CI [5.05, 5.36]) were afforded significantly more status than targets who were not (*M* = 4.64, 95% CI [4.46, 4.82], *t*(378.927) = 4.701, p < .001, *d* = .475).

**Fig 6:**
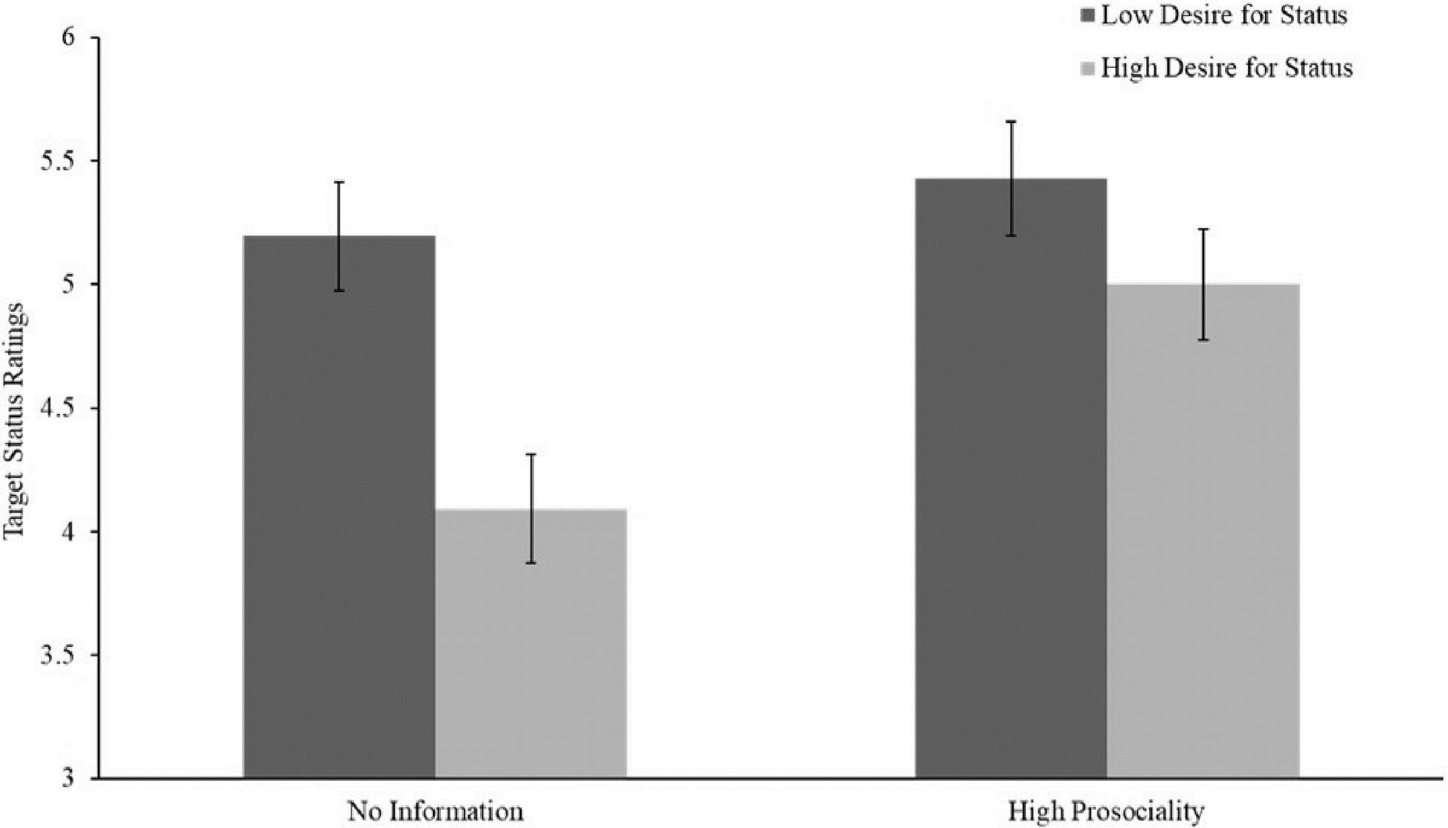
Moderation by prosociality. 95% CI shown.

A two-way between subjects ANOVA revealed a significant interaction between the desire for status and prosociality on status conferral, *F*(1, 383) = 8.668, *p* = .003. As predicted, the negative effect of desire for status on status conferral was stronger among targets whose prosocial tendencies were unknown than among targets who were described as being highly prosocial (see Fig 6). Among targets whose prosocial tendencies were unknown, targets described as having a high desire for status were given significantly less status (*M* = 4.09, 95% CI [3.89, 4.29]) than targets described as having a low desire for status (*M* = 5.20, 95% CI [4.93, 5.46], *t*(185.835) = 6.588, *p* < .001, *d* = .929). However, among targets described as being highly prosocial, while targets described as having a high desire for status were given less status (*M* = 5.00, 95% CI [4.78, 5.22]) than targets described as having a low desire for status (*M* = 5.42, 95% CI [5.22, 5.63], *t*(184.671) = 2.781, *p* = .036, *d* = .406), this difference was much smaller than among targets with unknown prosocial tendencies.

Similar to the findings on status, a two-way between subjects ANOVA revealed a significant interaction between the effects of desire for status and prosociality on prosociality perceptions, *F*(1, 383) = 8.526, *p* = .004, and competence perceptions, *F*(1, 383) = 5.412, *p* = .021. Among targets whose prosocial tendencies were unknown, targets described as having a high desire for status were seen as significantly less prosocial (*M* = 3.80, 95% CI [3.62, 3.98]) than targets described as having a low desire for status (*M* = 5.19, 95% CI [5.01, 5.37], *p* < .001, *d* = 1.24). However, among targets described as being highly prosocial, while targets described as having a high desire for status were seen as being significantly less prosocial (*M* = 4.65, 95% CI [4.46, 4.84]) than targets described as having a low desire for status (*M* = 5.49, 95% CI [5.30, 5.68], *p* < .001, *d* = .75), this difference was much smaller than among targets with unknown prosocial tendencies. Similarly, among targets whose prosocial tendencies were unknown, targets described as having a high desire for status were seen as significantly less competent (*M* = 5.17, 95% CI [5.00, 5.33]) than targets described as having a low desire for status (*M* = 5.73, 95% CI [5.57, 5.90], *p* < .001, *d* = .63). However, among targets described as being highly prosocial, while targets described as having a high desire for status were seen as being less competent (*M* = 5.79, 95% CI [5.62, 5.96]) than targets described as having a low desire for status (*M* = 5.96, 95% CI [5.78, 6.13], *p* = .55, *d* = .18), this difference was not significant and much smaller than among targets with unknown prosocial tendencies.

Replicating the negative effect of desire for status on prosociality perceptions, targets described as having a strong desire for status (*M* = 4.23, 95% CI [4.10, 4.36]) were seen as significantly less prosocial than targets described as having a weak desire for status (*M* = 5.34, 95% CI [5.21, 5.47], *p* < .001). As expected and serving as a manipulation check, targets described as being highly prosocial (*M* = 5.07, 95% CI [4.94, 5.20]) were seen as being significantly more prosocial than targets who were not (*M* = 4.50, 95% CI [4.37, 4.63], *p* < .001). Similarly, targets described as having a strong desire for status (*M* = 5.48, 95% CI [5.36, 5.60]) were seen as significantly less competent than targets described as having a weak desire for status (*M* = 5.85, 95% CI [5.72, 5.97], *p* < .001). Targets described as being highly prosocial (*M* = 5.87, 95% CI [5.75, 6.00]) were seen as being significantly more competent than targets who were not (*M* = 5.45, 95% CI [5.33, 5.57], *p* < .001).

To further understand the role of prosociality and competence in the affordance of status, we again used Preacher and Hayes’s bootstrapping procedure [42], 5000 resamples with replacement to derive a 95% bias-corrected confidence interval for the indirect effects of desire for status on status. Results from a simultaneous mediation with both perceived prosociality and competence included revealed an indirect effect for prosociality of -.5942 (95% CI:—.7340, -.4660), indicating a significant indirect effect. Perceived competence also had a significant indirect effect of -.1275 (95% CI:—.1974, -.0657). However, a pairwise contrast test of indirect effects indicated that the indirect effect of prosociality was significantly more negative, -.4667 (95% CI: -.6180, -.3238), than the indirect effect of competence. These findings suggest that participants afforded lower status to targets described as having a strong desire for status primarily because they viewed those targets as being less prosocial. These mediation analyses results are consistent with the mediation results from Studies 1 through 3a, suggesting perceived prosociality is the primary mediator of the effect.

These findings thus demonstrate that individuals with a strong (as opposed to weak) status motive were afforded lower status. However, the negative effect of strongly (vs. weakly) desiring status on status affordance was reduced when the target individual had a prosocial orientation (as compared to unknown prosocial orientation). These results—obtained using experimental manipulations which enable causal inference—again suggest that the desire for status harms status affordance, in part, because the desire for status is viewed as selfish and indicates lower prosociality.

## General discussion

### Summary of findings

Across five pre-registered studies (see Table 1 for summary), we found consistent evidence that individuals perceived as strongly desiring status were afforded less status than individuals perceived as weakly desiring status. This finding was due primarily to perceptions of prosociality; individuals seen as desiring more (versus less) status were given less status because they were seen as less prosocial and more selfish. In Studies 1a and 1b, we found in the workplace context that coworkers perceived as high in the desire for status were afforded lower status. Pinpointing the origins of causality, Studies 2, 3a, and 3b manipulated the perceived desire for status and replicated the negative effect of the desire for status on status affordance. In fact, we found that attributing the same behaviors to the desire for status, rather than the desire for achievement, resulted in significantly lower status afforded to the target (Study 2). Studies 3a and 3b found that when individuals were seen as possessing a strong desire for status for prosocial reasons (compared to selfish reasons), this mitigated the negative effect of being seen as desiring high status. Furthermore, Studies 1a, 1b, 2, 3a, and 3b find that prosociality mediates the relationship between desire for status and status affordance using bootstrapped mediation analyses, providing further support for our predicted mechanism.

**Table 1:** Summary of empirical evidence.

	N	Study type	Effect of desire for status on status	*P*-value	Effect	Evidence for indirect effect of prosociality
Study 1a	169	Correlational	Negative	.010	*β* = -.121	Yes
Study 1b	352	Correlational	Negative	< .001	*β* = -.141	Yes
Study 2	606	Experimental	Negative	< .001	*d* = .958	Yes
Study 3a	297	Experimental	Negative	< .001	*d* = 1.162	Yes
Study 3b	387	Experimental	Negative	< .001	*d* = .659	Yes
Total	1811					

Note. Cohen’s *d* reported for comparison to control condition for Study 2 and Study 3a.

The finding that the desire for status has a negative effect on status conferral might appear surprising. It would seem that whether someone *desires* to be highly respected is irrelevant to whether that person *deserves* to be highly respected; therefore, the degree to which a person desires status should not, at first blush, directly influence how much status they are conferred. However, it is important to keep in mind that status affordances are based on perceptions of competence and prosociality; if individuals are viewed as more selfish and less prosocial, they tend to be afforded less status. Therefore, the current findings are aligned with functional models of status. In addition, it seems that the desire for status, significantly affects status allocation in ways that may not have been previously accounted for in hierarchy and status research. The theory and data presented here provide a first step in better understanding the social consequences related to the desire for status and highlight an ironic effect behind the fundamental desire for status.

## Implications and future directions

Our findings have important implications to the growing literature on the attainment of status within groups [45–48]. Specifically, they speak to the characteristics that lead to attaining high or low status. In addition to demographic characteristics [49], personality traits [9], self-perceptions [32], and competence [50], it appears that individuals’ perceived motivation to have high status also plays an important role in determining their status. We also believe this finding primarily speaks on the prestige pathway of the dual pathways literature [5,6], but future research should consider the implications of this finding on the dominance pathways for the attainment of social rank. For example, future research could consider the extent to which one’s desire for status is perceived as reflecting vigorous or forceful pursuit of rank. If one’s strong desire for status is perceived as reflecting potentially coercive or forceful strategies, this finding may suggest a process that casts doubt on dominance models of rank pursuit. It would suggest that groups afford lower, rather than higher, status to individuals who pursue status too vigorously and forcefully. On the other hand, it may be the case that the desire for status is primarily perceived as a selfish interest to pursue prestige, and thus, only be of relevance to prestige pathways.

Our findings might seem to contradict previous studies that found a positive link between self-reported desire for status (rather than peer-rated) and status attainment. For example, some prior work found that people who report a stronger desire for status attain higher status (e.g., self-monitors; [9]). How could the same variable have both a positive and negative effect on status? Here it is important to distinguish peer-rated from self-reported desire for status. One possibility is that individuals have more insight into their own desire for status than do others. People may not be able to detect the desire for status in others that accurately. There is evidence suggesting that people actively try to hide their desire for status from others [29], which suggests people might not accurately perceive the desire for status in each other. If groups are indeed inaccurate in detecting the desire for status in members, this would suggest that groups actively try to punish those who have a high desire for status, as seen in our studies, but ultimately fail to accurately identify who they need to punish. Members who actually desire status more strongly (and report this desire in self-report surveys) might be able to attain high status because their desire is unknown to others. Further empirical testing of this idea is necessary.

Future research should also investigate other potential variables that may mediate the relationship between perceived desire for status and status affordances. Unexpectedly, many of our studies found that perceptions of competence play a mediating role in the relationship between the perceived desire for status and status affordances, even if its role was smaller than that for perceptions of prosociality. Specifically, in many (but not all) of our studies, targets perceived as desiring status more strongly were afforded less status in part because they were perceived as being less competent. As mentioned in the Introduction, we know of no prior theoretical or empirical work that would have suggested this finding. One possibility, however, is that the desire for status is viewed negatively, and therefore, when targets possess that desire, they are seen negatively along a variety of dimensions, akin to halo effects [51]. Future research should address this question and investigate other variables such as perceived threat or expectations of competition as potential mediators. For example, perceived threat has been found to predict support for social inequality (e.g., support for the status quo from members of high status groups; [52]). To the extent that such intergroup response to status threat extends to interpersonal status competition, threat perceptions driven by a target’s strong desire for status may also be driving the negative reactions to the target (e.g., lower status affordance).

While our studies have prioritized identifying the main effect of perceived desire for status on status attainment, another area needing additional research is on potential moderators of this effect. For example, we did not find consistent gender differences across our studies. However, prior research finds that targets of different genders are perceived differently for their desire for status [53], and that women are more likely to desire status (versus power) as a means to higher rank [10]. Future research should test whether women may face a stronger penalty for the desire for status.

Related, future research should examine whether ethnicity might moderate the effects we observed. Given that some minority group members face similar backlash as women when behaving assertively [53], it is possible that being perceived as strongly desiring status might lead to a similar backlash for those minority group members. Finally, it would be worthwhile to examine whether the effects we observed are stronger or weaker in other cultural contexts. We studied participants in the U.S. only, a very individualistic culture. Would being perceived as strongly desiring status incur even stronger social sanctions in more collectivistic and less individualistic cultures, which place relatively more emphasis on the welfare of the group rather than the individual?

## Supporting information

S1 Appendix (DOCX)
