# Supplementary material for: Be careful what you wish for: Individuals perceived to desire status are afforded less status
Source: PLoS One. 2024 Jun 25;19(6):e0304727. doi: 10.1371/journal.pone.0304727 (PMC11198812; doi:10.1371/journal.pone.0304727)
Supplement: S1 Appendix — (DOCX) [file pone.0304727.s001.docx]

**S1 Appendix**

**Study 2 Materials**

**Introduction to all conditions**

“Imagine you work with someone named [Kevin]/[Katherine]. As you have interacted with [Kevin]/[Katherine] over time, you have learned many things about who [he]/[she] is as a person. For example, [he]/[she] works very hard and is pleasant to deal with. [He]/[She] is diligent and has only missed a few days of work over the years. [He]/[She] has always come across as a dependable “team player” who is willing to make sacrifices for the organization’s success. [He]/[She] also rarely gets involved in confrontations with coworkers and seems pretty even-keeled emotionally, even in stressful situations.”

**Manipulations**

1. High desire for status condition (Kevin / Katherine)

You have also learned over time how much Kevin’s behavior is driven by his desire for status – that is, he is deeply concerned about being highly respected and admired by those around him, and being influential. He works hard in part because he cares so much about winning others’ esteem. While he is helpful with coworkers, he is particularly willing to help if others will find out about his sacrifices. For example, he is much more likely to work longer hours to help his team if he believes it will make others admire him more, and if he believes that his efforts are being seen. Even his happiness and self-esteem seem to depend on whether he feels respected and admired by his coworkers.

1. High desire for achievement condition (Kevin / Katherine)

You have also learned over time how much Kevin’s behavior is driven by his desire to achieve – that is, he is deeply concerned about accomplishing great things and performing as well as he can in all facets of life, including work. He works hard in part because he cares so much about achieving. While he is helpful with coworkers, he is particularly willing to help if it means he will feel a sense of accomplishment. For example, he is much more likely to work longer hours to help his team if he believes it will lead to higher performance and important accomplishments. His happiness and self-esteem seem to depend on whether he feels as though he is performing well at work.

1. Control condition (Kevin / Katherine)

You have also learned a bit about Kevin in his personal life, outside the workplace. He is still close with his family and stays in frequent contact with them. He likes to stay active outside of work but also enjoys his “downtime.” For example, on one weekend day he might go for a hike or work out at the gym, and on another day, he might go to the movies or read and putter around the house. He dates occasionally, and had a long-term girlfriend until about a year ago, but says he has not found the right person to spend the rest of his life with yet. His dream is to travel around the world one day and visit places that, so far, he has only read about in books or seen on the internet. He likes learning about new things and is curious about a lot of topics.

**Study 3a Materials**

**Introduction to all conditions:**

Please imagine that you are at the new employee orientation for a new job. You have been hired along with 3 other newly employed consultants (Kevin, Andrew, Daniel). Hired as a team of four, each of the four consultants, including you, have similar educational backgrounds, work experiences and qualifications. In general, you find that all four of you are similarly competent and hardworking people. The orientation goes smoothly, and you learn many more things about your new colleagues.

**Manipulations:**

1. High desire for status with low prosociality condition

In particular, you have learned throughout the orientation about Kevin’s personality. Specifically, you have learned how much Kevin’s behavior is driven by his desire for status – that is, he is deeply concerned about being highly respected and admired by those around him, and being influential. He works hard in part because he cares so much about winning others’ esteem.

You also learn in a conversation that Kevin’s strong desire for status roots from his belief that if he attains high status, he will be better equipped to help himself reach his own personal career goals. In other words, Kevin desires high status because he believes having high status will help him succeed as an individual and will bring him the trappings of having high status.

1. High desire for status with high prosociality condition

In particular, you have learned throughout the orientation about Kevin’s personality. Specifically, you have learned how much Kevin’s behavior is driven by his desire for status – that is, he is deeply concerned about being highly respected and admired by those around him, and being influential. He works hard in part because he cares so much about winning others’ esteem.

You also learn in a conversation that Kevin’s strong desire for status roots from his belief that if he attains high status, he will be better equipped to help his team and the company overall. In other words, Kevin desires high status because he believes having high status will help him be a high-contributing member of the team, allowing the team and the company to succeed.

1. Control condition

In particular, you have learned a bit about Kevin in his personal life, outside the workplace. He is still close with his family and stays in frequent contact with them. He likes to stay active outside of work but also enjoys his “downtime.” For example, on one weekend day he might go for a hike or work out at the gym, and on another day, he might go to the movies or read and putter around the house. He dates occasionally, and had a long-term girlfriend until about a year ago, but says he has not found the right person to spend the rest of his life with yet. His dream is to travel around the world one day and visit places that, so far, he has only read about in books or seen on the internet. He likes learning about new things and is curious about a lot of topics.

**Study 3b Materials**

**Desire for status manipulations:**

1. High desire for status condition


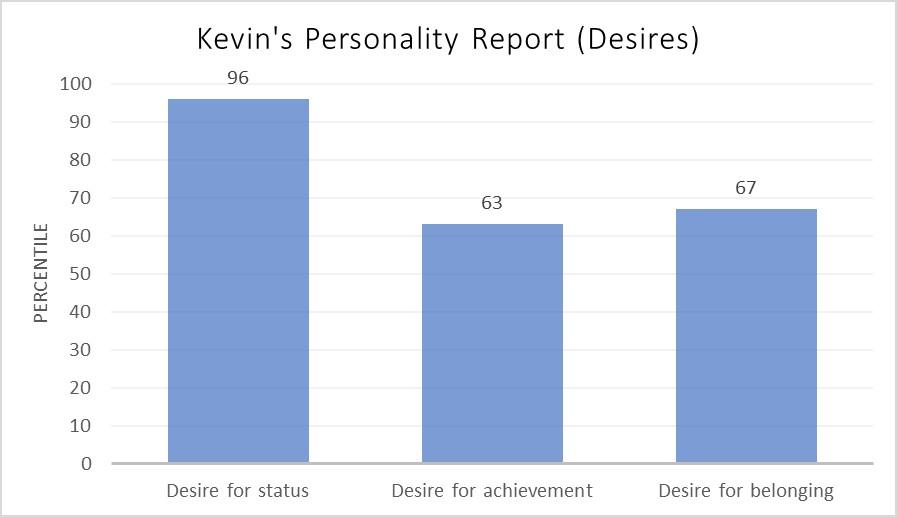


1. Low desire for status condition


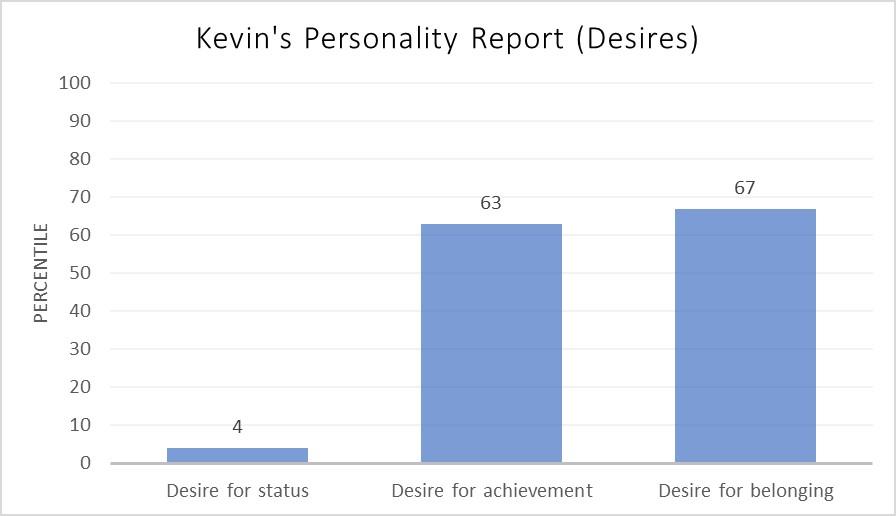


**Prosociality manipulations:**

1. High prosociality condition


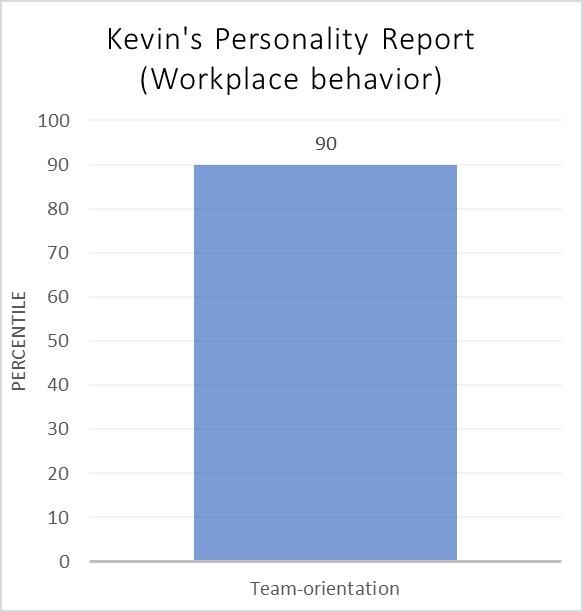


**Study 4 (Supplementary study)**

Study 4 aimed to test whether the negative effect of the perceived desire for status on status affordance is unique to the status motive. Specifically, we tested whether the negative effect of the desire for status on status affordance is stronger than is the effect of the desire for social acceptance on social acceptance. The desire for social acceptance was chosen as the comparison motive because it is also considered fundamental (Baumeister & Leary, 1995), and both desired outcomes – status and acceptance – relate to social attractiveness and depend on being perceived positively by others (Kenrick et al., 2010; Sheldon et al., 2001; Tay & Diener, 2011).

A pilot study (*N*=200; <https://aspredicted.org/blind.php?x=9tp4nf>) presented participants with the definitions and explanations for 9 fundamental motives (constructed from previous work on needs and motives, Sheldon, Elliot, Kim &, Kasser, 2001; Tay & Diener, 2011; Kenrick, Griskevicius, Neuberg, & Schaller, 2010), and asked, “How do you view the following motivations, whether these motivations are held by you or by others?” They rated each motive on a scale from -3 (“I view this motivation very negatively, as something people should not possess”) to 0 (“I view this motivation as neither good nor bad”) to 3 (“I view this motivation very positively, as something that is admirable to possess”). These results revealed that the desire for belongingness is (after the desire for status) the next most undesirable fundamental motive. Thus, the desire for belonging is a conservative comparison condition because it is also viewed as undesirable compared to the other fundamental desires.

Study 4 used an identical design to Study 3b to manipulate the desire for status, but also manipulated the desire for social acceptance using the same approach. We predicted that the desire for status would result in lower status affordance, but that the desire for social acceptance would not negatively influence judgments about the target’s social acceptance.

**Methods**

We preregistered the study on AsPredicted (<https://aspredicted.org/blind.php?x=y9dq85>).

**Participants and design.** A total of 800 participants were recruited from Amazon Mechanical Turk. The sample size of 800 allowed for 200 participants per cell. Participants were paid $.50 for an approximately 3-minute survey. The study had a 4-cell between-subjects design: 1) high desire for status target, 2) low desire for status target, 3) high desire for social acceptance target, and 4) low desire for social acceptance target. Twenty-six participants failed at least one of two preregistered attention checks, resulting in 777 total participants. All participants provided written informed consent. The recruitment period for this study began and ended on December 7^th^, 2020.

**Procedure.** Similar to Study 3b, participants were asked to imagine being a new hire attending a new employee orientation and learning about the personality of another new employee (Kevin). In the two desire for status conditions (high and low), participants either viewed a personality report describing that Kevin “placed at the 96th percentile on the desire for status” (high desire for status condition), or a personality report describing that Kevin “placed at the 4th percentile on the desire for status” (low desire for status condition); in both desire for status conditions, the target’s desire for social acceptance was held constant at the 50^th^ percentile. In the two desire for social acceptance conditions, participants either viewed a personality report describing that Kevin “placed at the 96^th^ percentile on the desire for social acceptance” (high desire for social acceptance), or a personality report describing that Kevin “placed at the 4^th^ percentile on the desire for social acceptance” (low desire for social acceptance); in both of the desire for social acceptance conditions, the target’s desire for status was held constant at the 50^th^ percentile. After reviewing the relevant materials, participants rated the target on the dimensions below.

**Materials (Survey).**

***Status conferred to target***. Status was measured by an identical three-item measure used in Studies 2, 3a and 3b (1 = *strongly disagree*, 7 = *strongly agree*; *α* = .86; *M* = 4.62, 95% CI [4.53, 4.71]).

**Social acceptance of target.** Social acceptance was measured by a four-item measure (“I would want to befriend Kevin”, “I would like Kevin”, “I would accept Kevin into the team”, “I would make sure Kevin feels included in the team”; adapted from (Anderson et al., 2006). The items showed internal consistency (*α* = .90) and were combined (*M* = 5.07, 95% CI [4.98, 5.15]).

**Results**

As predicted, we found a significant negative effect of desire for status on the affordance of status (Figure 6a). Targets described as having a high desire for status were afforded significantly less status (*M* = 4.23, 95% CI [4.02, 4.44]) than targets described as having a low desire for status (*M* = 5.02, 95% CI [4.88, 5.16], *t*(382) = 6.132, *p* < .001, *d* = -1.26). In contrast, we found a positive effect of the desire for social acceptance on social acceptance (Figure 6b). Targets described as having a high desire for social acceptance were accepted significantly more (*M* = 5.61, 95% CI [5.48, 5.75]) than targets described as having a low desire for social acceptance (*M* = 4.40, 95% CI [4.23, 4.57], *t*(391) = -10.903, *p* < .001, *d* = 1.10).

**[Figure 6a and 6b. Effect of desire for status or social acceptance on each desire specific dependent variables. 95% CI shown.]**

Because the two sets of conditions examined effects on different dependent variables (i.e., target status ratings and target social acceptance ratings), we could not test for an interaction. Therefore, we tested whether the effect of desire for status on status affordance was significantly more negative than the effect of desire for social acceptance on acceptance with a Fisher’s r-to-z transformation, followed by a Cohen and Cohen’s z-score comparison test (Cohen & Cohen, 1983; Raghunathan et al., 1996). The Fisher’s r-to-z transformations from the correlations between the desire for status conditions and the affordance of status (*r =* -.299, *n* = 384), and the correlations between the desire for social acceptance conditions and acceptance (*r* = .483, *n* = 393) resulted in *z =* -11.6, *p < .*001. The significant difference between the correlations suggests that the effect of desire strength on the relevant outcomes (i.e., status affordance for desire for status, and social acceptance for the desire for social acceptance) is significantly more negative for the desire for status than the desire for social acceptance (which in fact, showed a positive effect of desire strength).

**Discussion**

Study 4 provides evidence that the negative effect observed across our studies was unique to the desire for status. While being a similar fundamental human motive, having a high desire for social acceptance does not appear to have the same social costs as does the desire for status.
